# Generation and analysis of expressed sequence tags (ESTs) from cambium tissue cDNA libraries of contrasting genotypes of *Eucalyptus globulus* Labill

**DOI:** 10.1186/1753-6561-5-S7-P108

**Published:** 2011-09-13

**Authors:** Juan Pedro Elissetche, Alexis Salas-Burgos, Renan Garcia, Carola Iturra, Regis Teixeira, Jaime Rodriguez, Sofia Valenzuela

**Affiliations:** 1 Genomica Forestal SA, Barrio Universitario s/n, Centro de Biotecnología, Universidad de Concepción, Concepción, 403000, Chile

## 

In the present study, reported the generation and analysis of ESTs of cDNA libraries from cambium tissue of secondary xylem obtained of two genotypes of *Eucalyptus globulus* contrasting in wood density and pulp yield. The sequences were blasted and annotated to compare with genome and ESTs database of other plant species. The goal of study was to determinate wich genes was differentially expressed and compare levels of transcript, validated by qRT-PCR, in each genotype involved in wood formation, to explain the differences founding in wood traits of contrasting genotypes.Sequences obtained was 450,000 ESTs of which approximately 21,000 sequences showed homology with genes of different vascular plants, mainly *Vitis vinifera*, *Populus sp*, *Eucalyptus sp*, *Ricinus sp* . Moreover, it was determined that 265 genes differentially expressed in both genotypes, and 41 genes were directly involved in wood formation process (xylogenesis). Of the 41 differentially expressed genes could be determined that mainly correspond genes involved in lignin biosynthesis pathway which HCT, C3H, CAD, PAL, COMT and F5H and lignin polymerization like laccase and peroxidase. Otherwise we found genes involved in carbohydrate biosynthesis (cellulose and hemicellulose) among which Sussy, UDP glucose dehydrogenase, UDP-mannose dehydrogenase and β-xylosidase. Also we described genes involved in morphological characteristics of fiber such FLA, XTH and transcription factors such MYB and LIM related to fiber length, microfibrillar angle and extensibility of cell wall. Finally some of these genes were validated by the qRT-PCR technique to determine the level of transcripts in each genotype.

